# Insurance-related disparities in primary care quality among U.S. Type 2 diabetes patients

**DOI:** 10.1186/s12939-016-0413-x

**Published:** 2016-08-02

**Authors:** De-Chih Lee, Hailun Liang, Leiyu Shi

**Affiliations:** 1 Johns Hopkins Primary Care Policy Center, Baltimore, MD 21205 USA; 2 Department of Information Management, Da-Yeh University, No. 168, University Rd., Dacun, Changhua, 51591 Taiwan Republic of China; 3 Johns Hopkins Bloomberg School of Public Health, Baltimore, MD 21205 USA

**Keywords:** Insurance-related disparities, Primary care quality, Diabetes

## Abstract

**Background:**

This study explored insurance-related disparities in primary care quality among Americans with type 2 diabetes.

**Methods:**

Data came from the household component of the 2012 Medical Expenditure Panel Survey (MEPS). Analysis focused on adult subjects with type 2 diabetes. Logistic regressions were performed to investigate the associations between insurance status and primary care attributes related to first contact, longitudinality, comprehensiveness, and coordination, while controlling for confounding factors.

**Results:**

Preliminary findings revealed differences among three insurance groups in the first contact domain of primary care quality. After controlling for confounding factors, these differences were no longer apparent, with all insurance groups reporting similar primary care quality according to the four domains of interest in the study. There were significant differences in socioeconomic status among different insurance groups.

**Conclusion:**

This study reveals equitable primary care quality for diabetes patients despite their health insurance status. In addition to insurance-related differences, the other socioeconomic stratification factors are assumed to be the root cause of disparities in care. This research emphasizes the crucial role that primary care plays in the accessibility and quality of care for chronically ill patients. Policy makers should continue their commitment to reduce gaps in insurance coverage and improve access as well as quality of diabetic care.

## Background

Diabetes is one of the leading causes of deaths worldwide. According to the World Health Organization (WHO), around 1.5 million people worldwide died due to diabetes in 2012 [1]. In 2000, the prevalence of diabetes was about 171 million worldwide, and the WHO estimates that by 2030, the prevalence will rise to 366 million individuals [2]. In 2012, 9.3 % of the U.S. population had diabetes [3]. Diabetes is among the ten most expensive medical conditions in the U.S. [4]. The estimated diabetes costs in the U.S. in 2012 was $245 billion [3]. Diabetes is also associated with many health complications if preventive care and proper treatment is not received, including renal disease, non-traumatic lower limb amputations, blindness, and increased risk for cardiovascular disease and stroke [5].

Timely access to primary care and proper adherence to clinical treatment for diabetes can reduce the risk of health complications and improve long-term health outcomes for diabetes patients. Evidence suggests that insurance coverage can greatly improve diabetes patients’ access to care, having an impact on quality of care as well as health outcomes, especially when gaps and disparities are addressed [4–6].

Studies have shown a significant association between diabetes quality of care and insurance coverage. A study comparing the quality of diabetes care by insurance type in federally funded community health centers in the United States gives evidence of insurance-related disparities [4]. The results showed that those without insurance were least likely to meet the quality of care measures and that those with Medicaid had quality of care similar to those with no insurance [4]. The finding of lower quality and access to care for uninsured patients is supported by another study by Hu et al. This study noted that participants with private insurance or Medicare and Medicaid coverage were more likely to receive quality diabetes care than uninsured individuals [5]. A study by Booth et al. showed the universal drug coverage can help improve outcomes for diabetes patients of lower socioeconomic status [6].

While previous literature has uncovered insurance-related disparities in diabetes care and health outcomes, little exploration has been conducted on the relationship between insurance status and primary care quality among diabetic patients. This is important, as primary care has been proven to be effective in the management of diabetes [7–9]. The purpose of this study is to explore insurance-related differences in primary care quality – particularly, the cardinal attributes of first contact, longitudinality, comprehensiveness, and coordination [10] - among Americans with type 2 diabetes. First contact care means that care is first sought from the primary care provider when a new health or medical need arises. Longitudinality refers to the longitudinal use of a regular source of care over time, regardless of the presence or absence of disease or injury. Comprehensiveness refers to the availability of a wide range of services in primary care and their appropriate provision across the entire spectrum of types of needs for all but the most uncommon problems in the population by a primary care provider. Coordinated care is the linking of healthcare visits and services so that patients receive appropriate care for all their health problems, physical as well as mental [10, 11].

The unique contribution of this study lies in its enhanced generalizability by using a nationally-representative sample, up-to-date information on the topic, as well as empirical evidence for tracking the impact of the Affordable Care Act (ACA) on primary care system and the benefit for chronically ill patients.

## Methods

### Data

Data from the household component of the 2012 Medical Expenditure Panel Survey (MEPS) was used for this study. MEPS is a nationally representative survey of the US noninstitutionalized civilian population, composed of survey data of families and individuals, their medical providers, and employers. The annual data files are released with one common variance structure, which reflects the complex sample design of the MEPS. MEPS is supported by the Agency for Healthcare Research and Quality (AHRQ) [12]. The dataset used was the most currently released version at the time this study was conducted. The 2012 MEPS contained a total of 38,974 observations; the current study included respondents aged 18 and over who reported being told by a clinician that they had diabetes. We excluded respondents who had missing value for insurance status.

### Measures

The household component of MEPS collects detailed data on demographic characteristics, health conditions, health status, use of medical care services, charges and payments, access to primary care, satisfaction with care, health insurance coverage, income, and employment [12]. In this study, we selected measures of primary care attributes (dependent variables), types of health insurance (independent variable), and individual characteristics (covariates).

Following previous work conducted on primary care quality [10, 13, 14], we examined four cardinal attributes of primary care – first contact, longitudinality, comprehensiveness, and coordination – as dependent variables of interest. We selected eight measures from MEPS related to first contact attribute, which were having a usual source of care (USC) (yes, no); provider type of USC (facility, person/person in facility); provider specialty of USC (primary care, other); USC location (office, hospital); difficulty contacting USC by phone (not very difficult, very difficult); USC office hours on nights/weekends (yes, no); time to get to USC (<=30 min, >30 min); and difficulty getting to USC (not difficult, difficult). In terms of longitudinality, we used one measure of USC provider listening to patients (yes, no). For the attribute of comprehensiveness, we selected one question: going to USC for preventive health care (yes, no). Finally, two measures were selected for measuring the coordination, which were provider asking about other treatments (yes, no) and patient going to USC for referrals (yes, no).

We used Aday and Andersen’s access-to-care framework to select individual covariates that are potentially related to the primary care experience. Predisposing factors included: age (18–45, 46–64, above 64); sex (male, female); race/ethnicity (non-Hispanic White, non-Hispanic Black, Hispanic, Non-Hispanic Asian, Others); health insurance (public, private, no insurance); education (no degree, high school diploma, bachelor and higher degree, other); employment status (not employed, employed); income (<$20,000, $20,000–39,999, > = $40,000); and marital status (married, not married). Enabling factors included: metropolitan statistical area (MSA) (yes, no) and census region (northwest, Midwest, south, west). Need factors were: perceived health status (excellent/very good/good, fair/poor); perceived mental health status (excellent/very good/good, fair/poor); help with activities of daily living (ADL help screener) (yes, no); and help with instrumental activities of daily living (IADL help screener) (yes, no).

### Analysis

We performed all the data analyses by using Stata/SE 14.0. All the analyses accounted for both the design effect and the sampling weights by using svy command. Bivariate comparisons were performed between an individual’s insurance type and primary care measures related to first contact, longitudinality, comprehensiveness, and coordination. Chi-square tests were performed to determine whether there were differences between insurance groups in primary care quality. Logistic regressions were used to examine the association between insurance types and primary care measures, while controlling for individual covariates. We also performed bivariate comparison to show the variations in socioeconomic status (education, employment status and income) among types of insurance. We used standard errors, p-values, odds ratios, and 95 % confidence intervals to interpret statistical significance and effect size.

## Results

In 2012, it was estimated that more than 21.8 million Americans had type 2 diabetes. The majority of those were between the ages of 46 and 64 (44 %), and 65 and older (43 %). In terms of race/ethnicity, 60 % were non-Hispanic white, 17 % were Hispanic, 15 % were black, 5 % were Asian, and 3 % were others. Only 8 % were uninsured. 58 % were covered by private health insurance and 34 % were covered by public health insurance. Individuals with a high school diploma, the unemployed, and those with incomes below $20,000 accounted for over half of those with diabetes. About 41 % of diabetes cases were from the southern census region and 83 % were from urban areas. Table 1 shows additional information about the study population.

**Table 1:** Demographic and primary care characteristics for adults with diabetes

Personal Characteristics	Frequency	Weighted Frequency	Weighted %	Standard Error (SE)
Predisposing factors				
Age in years***				
18–45	403	2,924,379	13.41	0.86
46–64	1,164	9,561,753	43.84	1.5
Above 64	1,049	9,325,508	42.75	1.5
Sex**				
Male	1,230	11,156,923	51.15	1.2
Female	1,387	10,656,858	48.85	1.2
Race/Ethnicity***				
Non-Hispanic White	953	13,159,194	60.33	1.7
Non-Hispanic Black	683	3,368,274	15.44	1.1
Hispanic	743	3,592,967	16.47	1.4
Non-Hispanic Asian	171	1,012,791	4.64	0.55
Others	67	680,554	3.12	0.62
Health insurance***				
Private	1,260	12,567,988	57.61	1.5
Public	1,041	7,514,538	34.45	1.4
No insurance	316	1,731,254	7.94	0.65
Education***				
No Degree	373	2,135,699	20.92	1.3
High School Diploma	655	5,823,750	57.04	1.8
Bachelor and Higher Degree	193	1,686,156	16.51	1.3
Other	63	565,057	5.53	0.88
Employment status***				
Not employed	1,566	12,807,108	58.86	1.4
Employed	1,046	8,952,016	41.14	1.4
Income***		*		
< $20,000	1,500	10,985,633	50.39	1.4
$20,000–39,999	612	5,322,165	24.41	1
> = $40,000	504	5,493,176	25.2	1.3
Marital***				
No	1,245	9,458,394	43.36	1.2
Yes	1,372	12,355,387	56.64	1.2
Enabling factors				
MSA**		***		
No	387	3,870,681	17.75	1.6
Yes	2,229	17,940,960	82.25	1.6
Census region**		***		
Northeast	424	3,855,015	17.67	1.3
Midwest	449	4,705,831	21.57	1.3
South	1,109	8,893,429	40.77	1.4
West	634	4,357,367	19.98	1.1
Need factors				
Perceived health status ***		***		
Excellent/VG/Good	1,656	14,350,075	65.78	1.2
Fair/Poor	961	7,463,705	34.22	1.2
Perceived mental health status ***		***		
Excellent/VG/Good	2,203	18,510,339	84.86	0.93
Fair/Poor	414	3,303,441	15.14	0.93
ADL help screener ***		***		
No	2,457	20,608,010	94.47	0.64
Yes	160	1,205,770	5.53	0.64
IADL help screener ***		***		
No	2,362	19,750,771	90.54	0.72
Yes	255	2,063,009	9.46	0.72
Primary Care Attribute				
First Contact				
Have USC ***				
No	253	1,676,213	7.81	0.75
Yes	2,316	19,799,721	92.19	0.75
Provider type of USC ***				
Facility	1,140	8,879,718	44.85	1.71
Person/Person in facility	1,176	10,920,003	55.15	1.71
Provider specialty of USC				
Primary care	1,066	9,727,524	89.08	1.52
Other	110	1,192,479	10.92	1.52
USC location				
Office	1,664	15,321,478	77.48	1.44
Hospital	648	4,453,434	22.52	1.44
Difficulty in contacting USC by phone ***				
Not very difficult	2,096	17,845,798	93.95	0.71
Very difficult	139	1,148,599	6.05	0.71
USC has office hours nights/weekends ***				
No	1,412	12,252,897	68.91	1.44
Yes	670	5,528,129	31.09	1.44
How long it takes get to USC ***				
≤ 30 min	1,974	17,095,937	86.43	0.96
> 30 min	338	2,684,740	13.57	0.96
How difficult is it get to USC				
Difficulty	2,280	19,579,459	99.03	0.23
Not difficult	31	192,250	0.97	0.23
Longitudinality				
USC provider listens				
No	28	151,190	0.81	0.19
Yes	2,139	18,552,573	99.19	0.19
Comprehensiveness				
Goes to USC for preventive health care ***				
No	24	155,285	0.79	0.20
Yes	2,288	19,605,865	99.21	0.20
Coordination				
Provider asks about other treatments				
No	379	3,188,383	16.61	1.08
Yes	1,875	16,007,106	83.39	1.08
Goes to USC for referrals				
No	35	369,907	1.87	0.40
Yes	2,277	19,416,969	98.13	0.40

*p < 0.05, **p < 0.01, ***p < 0.001

When looking at first contact attributes of primary care among the study population by three insurance types, 69 % of uninsured reported having a usual source of care, compared to 94 % of privately-insured and 94 % of publicly-insured (*p* < .001). The uninsured overwhelmingly reported a facility to be their usual source of care (62 %) compared to people under private health insurance coverage (44 %) and under public insurance coverage (43 %) (*p* < .01). Hospitals accounted for 31 % of USC locations among uninsured, 26 % among publicly-insured, and only 20 % among privately-insured (*p* < .01). About 1 % of privately-insured, 2 % of publicly-insured and 1 % of uninsured reported not difficult in getting to USC (*p* < .01). When looking at the other measures in first contact as well as the measures regarding the longitudinality, comprehensiveness and coordination attributes of primary care, no additional significant differences were found. Additional findings are presented in Table 2.

**Table 2:** Primary care characteristics for adults with diabetes, by insurance status

	Insurance											
	Private, % (SE)				Public, % (SE)				Uninsured, % (SE)			
Primary Care Attribute	Freq	Weighted Frequency	Weighted %	SE	Freq	Weighted Frequency	Weighted %	SE	Freq	Weighted Frequency	Weighted %	SE
First Contact												
Have USC ***												
No	86	702,336	5.65	0.9	68	457,288	6.21	1.1	99	516,590	30.84	3.8
Yes	1,159	11,732,932	94.35	0.9	946	6,908,250	93.79	1.1	211	1,158,539	69.16	3.8
Provider type of USC **												
Facility	542	5,184,360	44.19	2.2	454	2,982,421	43.17	2.2	144	712,937	61.54	4.7
Person/Person in facility	617	6,548,572	55.81	2.2	492	3,925,828	56.83	2.2	67	445,603	38.46	4.7
Provider specialty of USC												
Primary care	562	5,821,805	88.9	2.1	446	3,512,108	89.46	2	58	393,611	88.33	4.5
Other	55	726,767	11.1	2.1	46	413,720	10.54	2	9	51,992	11.67	4.5
USC location **												
Office	875	9,386,435	80.07	1.8	656	5,139,253	74.48	2	133	795,790	69.09	4.4
Hospital	282	2,336,115	19.93	1.8	289	1,761,217	25.52	2	77	356,102	30.91	4.4
Difficulty in contacting USC by phone												
Not very difficult	1,060	10,580,584	94.5	1	861	6,263,976	93.5	1.1	175	1,001,238	91.13	2.1
Very difficult	55	615,910	5.5	1	58	435,224	6.5	1.1	26	97,466	8.87	2.1
USC has office hours nights/weekends												
No	679	7,168,320	67.41	2	603	4,379,029	71.62	2.1	130	705,548	68.31	4.4
Yes	373	3,465,310	32.59	2	236	1,735,547	28.38	2.1	61	327,272	31.69	4.4
How long it takes get to USC												
≤ 30 min	1,014	10,313,811	87.9	1.4	792	5,835,272	84.49	1.4	168	946,853	82.94	3.1
> 30 min	145	1,419,120	12.1	1.4	153	1,070,859	15.51	1.4	40	194,761	17.06	3.1
How difficult is it get to USC **												
Difficulty	1,152	11,671,618	99.49	0.24	923	6,778,351	98.25	0.43	205	1,129,490	98.94	0.69
Not difficult	6	59,276	0.51	0.24	22	120,850	1.75	0.43	3	12,124	1.06	0.69
Longitudinality												
USC provider listens												
No	9	65,133	0.6	0.26	16	76,154	1.16	0.3	3	9,903	0.85	0.51
Yes	1,057	10,880,003	99.4	0.26	889	6,511,449	98.84	0.3	193	1,161,121	99.15	0.51
Comprehensiveness												
Goes to USC for preventive health care												
No	8	61,016	0.52	0.21	11	79,061	1.15	0.42	5	15,208	1.32	0.69
Yes	1,150	11,652,671	99.48	0.21	934	6,820,140	98.85	0.42	204	1,133,054	98.68	0.69
Coordination												
Provider asks about other treatments												
No	183	1,833,937	16.09	1.5	154	1,155,387	17.3	1.6	42	199,060	17.83	3.3
Yes	947	9,566,148	83.91	1.5	768	5,523,532	82.7	1.6	160	917,426	82.17	3.3
Goes to USC for referrals												
No	18	194,748	1.66	0.5	13	161,616	2.34	0.81	4	13,543	1.18	0.62
Yes	1,141	11,538,183	98.34	0.5	933	6,746,634	97.66	0.81	203	1,132,151	98.82	0.62

*p < 0.05, **p < 0.01, ***p < 0.001

After controlling for individual’s predisposing, enabling, and needs factors, including race/ethnicity, insurance, age, gender, employment status, education, marital status, income, health status, mental health status, having an ADL or IADL screener, MSA and region, the differences found in Table 2 were no longer significant. Table 3 shows the results of logistic regressions associating health insurance status with primary care quality according to the four domains of primary care. Model 1 shows the unadjusted odds ratios expressed as the odds of each primary care attribute among each health insurance group compared with privately-insured. Similar to the findings from Table 2, the uninsured were less likely to have USC compared with people under private insurance coverage (OR = 0.134, *P* < .001). The uninsured were more likely to report a facility to be their usual source of care (OR = 2.021, *P* < .01) and were less likely to report office as their USC locations than privately-insured (OR = 0.556, *P* < .001). The publicly-insured were also less likely to report an office as their USC location than privately-insured (OR = 0.726, *P* < .05). The publicly-insured were 3.511 times more likely to have difficulties in getting to their USC than privately-insured.

**Table 3:** Logistic regressions: primary care characteristics for adults with diabetes, insurance status

	Odds Ratio (95 % CI)			
	Model 1		Model 2	
Primary Care Attribute	Public vs. Private	Uninsured vs Private	Public vs. Private	Uninsured vs Private
Have USC				
Yes	0.904 (0.557 1.467)	0.134 *** (0.081 0.222)	0.831 (0.484 1.428)	0.186 *** (0.103 0.337)
No	1	1	1	1
Provider type of USC				
Facility	0.960 (0.774 1.189)	2.021 ** (1.317 3.100)	0.794 (0.598 1.055)	1.352 (0.863 2.118)
Person/Person in facility	1	1	1	1
Provider specialty of USC				
Primary care	1.060 (0.614 1.828)	0.945 (0.373 2.397)	1.072 (0.550 2.087)	1.056 (0.373 2.989)
Other	1	1	1	1
USC location				
Office	0.726 * (0.552 0.956)	0.556 ** (0.366 0.845)	0.893 (0.628 1.270)	0.797 (0.506 1.254)
Hospital	1	1	1	1
Difficulty in contacting USC by phone				
Very difficult	1.194 (0.683 2.085)	1.672 (0.896 3.120)	0.875 (0.420 1.821)	1.234 (0.652 2.337)
Not very difficult	1	1	1	1
USC has office hours nights/weekends				
Yes	0.820 (0.622 1.080)	0.960 (0.613 1.501)	0.915 (0.656 1.276)	0.936 (0.576 1.521)
No	1	1	1	1
How long it takes get to USC				
≤ 30 min	0.750 (0.524 1.073)	0.669 (0.402 1.113)	0.914 (0.598 1.396)	0.867 (0.491 1.530)
> 30 min	1	1	1	1
How difficult is it get to USC				
Difficulty	3.511 ** (1.369 9.006)	2.113 (0.367 2.160)	1.011 (0.301 3.392)	0.905 (0.111 7.392)
Not difficult	1	1	1	1
Longitudinality				
USC provider listens				
Yes	0.512 (0.187 1.398)	0.702 (0.159 3.102)	0.742 (0.235 2.341)	1.926 (0.416 8.927)
No	1	1	1	1
Comprehensiveness				
Goes to USC for preventive health care				
Yes	0.452 (0.149 1.372)	0.390 (0.104 1.460)	0.730 (0.191 2.790)	0.478 (0.107 2.136)
No	1	1	1	1
Coordination				
Provider asks about other treatments				
Yes	0.917 (0.679 1.238)	0.884 (0.541 1.443)	0.925 (0.646 1.325)	0.887 (0.525 1.497)
No	1	1	1	1
Goes to USC for referrals				
Yes	0.705 (0.278 1.784)	1.411 (0.407 4.888)	0.543 (0.149 1.974)	0.932 (0.233 3.735)
No	1	1	1	1

Notes: Model 2 adjusted for race/ethnicity, insurance, age, gender, employment status, education, marital, income, health status, mental health status, ADL screener, IADL screener, MSA, and region

*p < 0.05, **p < 0.01, ***p < 0.001

Model 2 shows the results of multivariate logistic regressions. Odds ratios have been adjusted for individuals’ covariates that are potentially related to the primary care experience. After accounting for the individuals’ predisposing, enabling and need factors, the significant differences in primary care quality, which were found in Model 1, were no longer apparent. More specifically, only one insurance group, the uninsured, was still associated with lower odds in having USC (OR = 0.186, *P* < .001). No negative associations were found between privately-insured and primary care quality. The significant associations found in Model 1, between uninsured and higher odds of reporting a facility as their USC provider, and between uninsured and lower odds of reporting an office as their USC location, were no longer statistically significant after controlling for the confounding factors. In terms of the longitudinality, comprehensiveness and coordination attributes, there was no statistically significant association found between insurance types and primary care quality.

Table 4 shows the variations in socioeconomic status (education, employment status and income) among three types of insurance. Sixty-two percent of privately-insured reported having a high school diploma, compared to 50 % of publicly-insured and 50 % of uninsured (*p* < .001). Most of the publicly-insured were unemployed (89 %) compared to people under private health insurance coverage (43 %) or uninsured (42 %) (*p* < .001). Thirty-three percent of the privately-insured reported their income level as below $20,000, compared to 77 % of the publicly-insured and 64 % of the uninsured (*p* < .001).

**Table 4:** Socioeconomic status (SES) for adults with diabetes, by insurance status

	Private		Public		Uninsured	
SES	Weighted %	SE	Weighted %	SE	Weighted %	SE
Education ***						
No Degree	9.35	1.28	36.92	2.73	35.34	4.76
High School Diploma	62.31	2.43	49.86	3.05	49.94	5.46
Bachelor and Higher Degree	21.17	1.96	9.85	1.63	11.26	3.07
Other	7.17	1.28	3.37	1.13	3.47	1.65
Employment status ***						
Not employed	43.28	2.03	88.73	1.20	41.93	3.81
Employed	56.72	2.03	11.27	1.20	58.07	3.81
Income ***						
< $20,000	32.83	1.7	76.68	2.04	64.40	4.21
$20,000–39,999	29.30	1.46	16.73	1.61	21.98	3.26
> = $40,000	37.88	1.80	6.59	1.19	13.62	3.36

*p < 0.05, **p < 0.01, ***p < 0.001

## Discussion

The study used nationally-representative MEPS data to explore the presence of disparities in quality of primary care, and to build on past research investigating whether insurance differences in quality of care persist in an effort to eliminate health disparities over the years. The unadjusted results revealed differences in primary care quality among different insurance groups across measures in the first contact attribute. After accounting for the individuals’ predisposing, enabling and need factors, the significant differences were no longer apparent. Our study suggests that equitable primary care quality was received by diabetes patients despite their health insurance status and implies the crucial role that primary care plays in providing a more equitable level of care for patients with chronic disease.

Previous studies suggests that there were insurance-related disparities in access to primary care, the medical management of chronic illness, health care qualities and health outcomes [15–17]. However, with ACA providing a solid foundation for expansions in health insurance coverage and strengthening the U.S. primary care system [18], health care disparities have been narrowed among groups with different insurance statuses. The ACA spurred major expansions in health insurance coverage, with some of the biggest gains from the federally operated marketplace and in states that expanded eligibility for their Medicaid programs. About 6.7 million people enrolled in health plans sold through the ACA’s marketplaces in 2014 [19]. Nearly 10 million people have newly enrolled in Medicaid since October 2013 [20]. Moreover, multiple provisions were included in the ACA for improving primary care, such as the support for innovation in primary care delivery, increasing Medicaid and Medicare payments to primary care providers, and investing in primary care workforce development. For chronic disease patients, the ACA advances the “medical home” concept for Medicaid patients with chronic conditions. Starting in 2011, millions of Medicaid patients with chronic conditions could have a health home to help them manage their condition. Such reforms in health insurance coverage and primary care provide a solid foundation for strengthening the U.S. primary care system, and have a positive impact on patient’s care, especially for chronically ill patients [18]. Policy makers should continue their commitment to target vulnerable groups, such as elderly, poor, and/or medically underserved populations, and reduce gaps in insurance coverage in further and therefore ultimately improving access and quality of care.

This study has several limitations. First, it is difficult to make causal inferences due to the secondary nature of the dataset. Second, MEPS data on primary care relies on household respondents’ self-report, which is subject to recall bias. Third, the study only included measures regarding primary care experience reported by the patients, rather than their health outcomes. Further studies may include more health outcomes measures, such as clinical performance indicators, to evaluate the impact of health insurance on diabetic care outcomes. Fourth, our results showed there were significant differences in socioeconomic status among major types of health insurance. The analysis could be improved to present analyses that characterize the degree to which each type of SES explained the insurance-quality associations in the unadjusted models, such as by using a hierarchical modeling approach. Lastly, our primary care measures were operationalized from MEPS rather than the investigator-initiated, which preclude the examination of all the major measures of primary care, especially with regard to measures for the logitudinality and comprehensiveness attributes.

Despite these shortcomings, this study demonstrates important findings to the field and could contribute to improving primary care for diabetic patients. This study reveals equitable primary care quality for diabetes patients despite their health insurance status. In addition to insurance-related differences, other socioeconomic stratification factors, such as the inequality income, education, and occupation, are assumed to be the root cause of disparities in care and population health [21]. Future efforts are needed to investigate both insurance-related and SES-based disparities in healthcare, to identify the major mediators of differences in quality of care. The bulk of the evidence suggests that equitable primary care eliminates racial and ethnic disparities [22–25]. Next steps and future directions should be undertaken to examine the role of primary care in improvements in the management of chronic diseases by reducing both insurance and SES-based disparities. In conclusion, the causes of disparities in diabetes care are complex and include societal issues such as lower SES status and poor access to health care. The affordable care act has improved accessibility and affordability of health care. To further improve the quality and equity of primary care for diabetes patients, a number of policy changes could potentially make a positive contribution, such as encouraging new models of care for pre-diabetes and diabetes patients, and raising reimbursement levels for primary care providers who deliver evidence-based diabetes prevention and care.

## Conclusions

This study reveals equitable primary care quality for diabetes patients despite their health insurance status. In addition to insurance-related differences, the other socioeconomic stratification factors are assumed to be the root cause of disparities in care. This research emphasizes the crucial role that primary care plays in the accessibility and quality of care for chronically ill patients. Policy makers should continue their commitment to reduce gaps in insurance coverage and improve access as well as quality of diabetic care.

## Abbreviations

ACA, Affordable Care Act; ADL, Activities of daily living; AHRQ, Agency for Healthcare Research and Quality; IADL, Instrumental activities of daily living; MEPS, Medical Expenditure Panel Survey; MSA, Metropolitan statistical area; USC, Usual source of care; WHO, World Health Organization
